# Pullularins E and F, Two New Peptides from the Endophytic Fungus *Bionectria ochroleuca* Isolated from the Mangrove Plant *Sonneratia caseolaris*

**DOI:** 10.3390/md10051081

**Published:** 2012-05-18

**Authors:** Weaam Ebrahim, Julia Kjer, Mustapha El Amrani, Victor Wray, Wenhan Lin, Rainer Ebel, Daowan Lai, Peter Proksch

**Affiliations:** 1 Institute of Pharmaceutical Biology and Biotechnology, Heinrich-Heine University, Universitaetsstrasse 1, D-40225 Duesseldorf, Germany; Email: weel-001@uni-duesseldorf.de or weaamnabil@mans.edu.eg (W.E.); jacob.julia@web.de (J.K.); mustapha.elamrani@uni-duesseldorf.de (M.E.A.); 2 Department of Pharmacognosy, Faculty of Pharmacy, Mansoura University, Mansoura 35516, Egypt; 3 Helmholtz Centre for Infection Research, Inhoffenstraße 7, Braunschweig D-38124, Germany; Email: victor.wray@helmholtz-hzi.de; 4 National Research Laboratories of Natural and Biomimetic Drugs, Health Science Center, Peking University, Beijing 100083, China; Email: whlin@bjmu.edu.cn; 5 Marine Biodiscovery Centre, University of Aberdeen, Meston Walk, Aberdeen, Scotland AB24 3UE, UK; Email: r.ebel@abdn.ac.uk

**Keywords:** mangrove plants, *Sonneratia*, endophytes, *Bionectria*, peptide, structure elucidation, Marfey’s method

## Abstract

Chemical investigation of the EtOAc extract of the endophytic fungus *Bionectria ochroleuca*, isolated from the inner leaf tissues of the plant *Sonneratia caseolaris* (Sonneratiaceae) from Hainan island (China), yielded two new peptides, pullularins E and F (**1** and **2**) together with three known compounds (**3**–**5**). The structures of the new compounds were unambiguously determined on the basis of one- and two-dimensional NMR spectroscopy as well as by high-resolution mass spectrometry. The absolute configurations of amino acids were determined by HPLC analysis of acid hydrolysates using Marfey’s method. The isolated compounds exhibited pronounced to moderate cytotoxic activity against the mouse lymphoma cells (L5178Y) with EC_50_ values ranging between 0.1 and 6.7 µg/mL.

## 1. Introduction

Endophytic fungi are a fascinating source of new natural products which are of great potential for medicinal and agricultural applications [1,2,3,4]. Mangrove plants live in tropical and subtropical forests and share the ability to grow in estuarine and coastal environments. They are open systems with respect to both energy and biomatter and thus couple upland terrestrial and coastal estuarine ecosystems [5]. Mangrove-derived endophytes are attracting significant attention due to their potential of producing novel metabolites. Examples of those bioactive metabolites are the depsidone-type compound paeciloxocin A isolated from *Paecilomyces* sp., isolated from the bark of an unidentified mangrove from the Taiwan Strait, which was found to have potent cytotoxic activity [6]. Penicinoline was separated from a *Penicillium* sp., isolated from the bark of the mangrove *Acanthus ilicifolius* (Acanthaceae) collected from the South China Sea; it showed *in vitro* cytotoxic activity against 95-D and HepG2 cell lines [7]. Chloropupukeanolide A from *Pestalotiopsis fici*, obtained from branches of an unidentified mangrove in the suburb of Hangzhou, China, exhibited anti-HIV1 activity when tested *in vitro* [8].

During our ongoing search for new bioactive metabolites from plant-derived endophytes [9,10,11], we isolated an endophytic *Bionectria ochroleuca* strain from leaf tissues of the mangrove plant *Sonneratia caseolaris* (Sonneratiaceae), collected at Hainan Island in China.

Natural products of fungi belonging to the genus *Bionectria* were only rarely studied so far. A literature survey showed that *Bionectria* sp. yielded bionectriol A and TMC-151 which are both metabolites of polyketide origin [12,13]. Furthermore, piperazine derivatives bionectines A–C, glioperazine B and C and verticillin G were also reported from *Bionectra byssicola* [14,15,16]. In the present study we provide a comprehensive analysis of natural products produced by *Bionectria ochroleuca* and report on two new peptides designated pullularins E (**1**) and F (**2**), and two known congeners pullularins A (**3**) and C (**4**) [17], in addition to the fungal epipolythiodioxopiperazine metabolite verticillin D (**5**) [18].

## 2. Results and Discussion

The crude ethyl acetate extract of *Bionectria ochroleuca* cultured on solid rice medium, was taken to dryness and partitioned between *n*-hexane and 90% methanol. The 90% methanol fraction was chromatographed over different stationary phases (silica gel and Sephadex LH-20). Final purification by preparative reversed-phase HPLC afforded five compounds whose structures were elucidated by high resolution ESI mass spectrometry (HRESIMS) and NMR spectroscopy.

Pullularin E (**1**) was obtained as a white powder. Its UV maxima at 227 and 277 nm concurred with its colorless appearance. The molecular formula C_42_H_57_N_5_O_8_ was derived from the HRESIMS exhibiting a peak at *m/z* 760.4264 (calcd. for C_42_H_58_N_5_O_8_ 760.4285). After NMR data collection using CDCl_3_, the HRESIMS of this compound showed different pseudomolecular peaks at *m/z* 794.3881 (100%, [M + H]^+^), and 796.3866 (32%, [M + 2 + H]^+^) indicating the molecular formula C_42_H_56_ClN_5_O_8_, containing one chlorine atom. Therefore, compound **1** was totally transformed to a chloro-derivative (**1a**). Compound **1a** was re-measured in DMSO-*d*_6_ to get better resolution of the NMR spectra. Extensive analysis of the NMR data of **1a** and comparison with those reported for pullularins A (**3**) and C (**4**) [17] indicated a close structural relationship of **1a** with the latter depsipeptides. The number of hydrogen and carbon atoms observed in the ^1^H and ^13^C NMR spectra of **1a** was in agreement with the molecular formula, indicating that **1a** is a hexadepsipeptide composed of one 2-hydroxycarboxylic acid moiety and five amino acid residues. Correspondingly, one ester carbonyl carbon (δ_C_ 169.5) and five amide carbonyl carbons (δ_C_ 168.1, 165.4, 171.8, 173.8 and 168.0) were discernible. Since only two -NH proton signals (δ_H_ 9.53 and 8.89) and two *N*-methyl groups (δ_H_ 2.93 and 2.40) were observed in the ^1^H NMR spectrum of **1a**, the fifth amino acid was assumed to represent proline. This assumption was corroborated by analysis of the TOCSY spectrum, which in addition allowed for assigning the spin systems and furthermore hinted at the presence of an *O*-isoprenyl residue. The positions of the *N*-methyl groups, the prenyl residue, the sequence of the amino acid residues and the 2-hydroxycarboxylic acid were established by extensive analysis of the HMBC and ROESY data (Table 1).

**Table 1:** NMR data of **1a** in DMSO-*d*_6_*^a^* and key ^1^H–^1^H COSY, HMBC, and ROESY correlations.

Position	δ_C_ *^b^*	δ_H_ mult. (*J* Hz)*^c^*	COSY	HMBC (H→C)	ROESY
***O*-isoprenyl-Tyr**					
1 (C=O)	168.1 C *^d^*				
2	51.2 CH	4.72 ddd (4.4, 8.8, 8.8)	3, NH	1, C=O (*N*-Me-lle)	*N*-CH_3_ (*N*-Me-Ala)
3	36.9 CH_2_	2.95 dd (9.8, 13.8)	2, 3b	1, 2, 4, 5, 9	
		2.41 m	2, 3a	1, 2, 4, 5, 9	
4	130.7 C				
5, 9	130.2 CH	7.04 d (8.6)	6, 8	3, 4, 6, 7	
6, 8	114.4 CH	6.83 d (8.6)	5, 9	5, 7	
7	156.2 C				
1′	69.7 CH_2_	4.18 ddd (1.8, 6.0, 10.6)	2′, 1′b	2′, 3′, 7	
		4.15 ddd (0.8, 7.3, 10.6)	2′, 1′a		
2′	63.0 CH	4.86 ddd (2.1, 6.5, 6.5)	1′	1′, 3′, 4′, 5′	
3′	141.3 C				
4′	116.5 CH_2_	5.20 s	4′b, 5′ (weak)	2′, 3′, 5′	
		5.04 s	4′a, 5′ (weak)	2′, 5′	
5′	17.6 CH_3_	1.80 s	4′ (weak)	2′, 3′, 4′	
NH		9.53 d (8.7)	2	1, 2, C=O (*N*-Me-lle)	2 (*N*-Me-lle)
***N*-Me-Ala**					
1 (C=O)	169.5 C				
2	58.4 CH	3.69 q (6.7)	3	1, 3, N-CH_3_, C=O (*O*-isoprenyl-Tyr)	*N*-CH_3_
3	13.0 CH_3_	1.05 d (6.6)	2	1, 2	*N*-CH_3_
*N*-CH_3_	35.8 CH_3_	2.93 s		2, C=O (*O*-isoprenyl-Tyr)	2, 3, 2 (*O*-isoprenyl-Tyr)
**3-Ph-Lac**					
1 (C=O)	165.4 C				
2	71.5 CH	5.50 dd (5.1, 8.7)	3	1, 3, 4, C=O (*N*-Me-Ala)	5 (Pro)
3	35.8 CH_2_	3.11 dd (8.8, 13.0)	2, 3b	1, 2, 4, 5,9	
		2.81 dd (4.9, 13.0)	2, 3a	1, 2, 4, 5,9	
4	136.4 C				
5, 9	129.5 CH	7.19 (overlapped)	6, 8	3, 6, 7	
6, 8	128.4 CH	7.26 t (7.5)	5, 7, 9	4, 5, 7	
7	126.6 CH	7.19 (overlapped)	6, 8	5, 6	
**Pro**					
1 (C=O)	171.8 C				
2	58.4 CH	4.31 dd (1.8, 8.6)	3	3, 4, 5	NH (Ala)
3	29.1 CH_2_	2.03 m	2, 3b, 4	1	
		1.68 m	2, 3a, 4	4	
4	23.8 CH_2_	1.82 m	3, 5		
5	45.8 CH_2_	3.75 ddd (3.0, 8.7, 8.7)	4, 5b	3	2 (3-Ph-Lac)
		3.21 q (8.5)	4, 5a	4	2 (3-Ph-Lac)
**Ala**					
1 (C=O)	173.8 C				
2	44.4 CH	4.65 m	3, NH	1, 3, C=O (Pro)	
3	17.2 CH_3_	1.21 d (6.6)	2	1, 2	*N*-CH_3_ (*N*-Me-lle)
NH		8.89 d (4.3)	2	1, 1 (Pro), 3	3, 2 (Pro), 3b (Pro)
***N*-Me-lle**					
1 (C=O)	168.0 C *^d^*				
2	64.8 CH	4.53 d (10.8)	3	1, 3, 3-CH_3_, N-CH_3_, C=O (Ala)	NH (*O*-isoprenyl-Tyr)
3	32.0 CH	2.00 m	2, 4, 3-CH_3_		
4	24.3 CH_2_	1.22 m	3, 5		
		0.90 m			
5	11.8 CH_3_	0.89 t (6.7)	4	3, 4	
3-CH_3_	15.9 CH_3_	0.91 d (6.4)	3	2, 3, 4	
*N*-CH_3_	28.4 CH_3_	2.40 s		2, C=O (Ala)	3 (Ala)

*^a^* Measured at 600 (^1^H) and 150 (^13^C) MHz; *^b^* Multiplicities were deduced from DEPT and HSQC experiments; *^c^* The assignments for methylene protons were referred as “a” in upper row, and “b” in the next row; *^d^* Assignments within a column maybe interchanged.

The 2-hydroxycarboxylic acid was assigned to be 3-phenyllactic acid (3-Ph-Lac), on the basis of an oxymethine (δ_C_ 71.5) attached to a methylene at δ_C_ 35.8, which in turn was adjacent to a monosubstituted phenyl group. Extensive analysis of the NMR data showed that the serine residue in the known compound **3** was replaced by an alanine residue in **1a**, as evidenced by the upfield shifted signals at δ_H_ 4.65 of the α-proton and at δ_H_ 1.21 of the aliphatic methyl group in the alanine residue. Its -NH group at δ_H_ 8.89 showed correlations to the methyl group and the carbonyl groups of proline, indicating that both amino acids were adjacent.

The NMR spectra of **1a** differed from those of **3** and **4** furthermore by the nature of the isoprene unit. Instead of two olefinic methyl groups as present in compounds **3** and **4** only one signal was found for **1a** at δ_H_ 1.80, sharing a COSY cross peak with the methylene function CH_2_-4′ at (δ_H_ 5.20 and 5.04). The olefinic methyl proton signal also correlated with a quaternary carbon C-3′ at δ_C_ 141.3 and a methine carbon C-2′ at δ_C_ 63.0 in the HMBC spectrum. Moreover, a chlorine was assumed to be attached at C-2′, based on the chemical shifts of CH-2′ (δ_C_ 63.0, δ_H_ 4.86). Thus, **1a** featured an *O*-isopentenyl moiety as isoprene substituent instead of an *O*-dimethylallyl residue as present in **3** and **4**.

For the determination of the stereochemistry of the amino acid residues, the so-called advanced Marfey’s method was employed [19]. After hydrolysis of **1a** in 6 M HCl for 24 h, the hydrolyzate was transformed to diastereomeric reaction products by adding the reagent FDAA. The reaction products thus obtained were submitted to analytical HPLC and LCMS. Moreover, the commercially available amino acids tyrosine, *N*-methyl-isoleucine, alanine, proline and *N*-methyl-alanine were used as authentic standards, both as the respective L-enantiomers and as racemates, and subjected to similar conditions for the derivatization and analysis. By comparison of the retention times of the reaction products of the hydrolyzate and the amino acid standards, the configuration of the respective amino acid was determined. As tyrosine lost its isoprenyl residue during hydrolysis, it was not necessary to provide *O*-isoprenyl tyrosine derivatives. On this basis, the configurations of the amino acids in **1a** were determined as L-Tyr, *N*-Me-L-Ile, L-Ala, L-Pro and *N*-Me-L-Ala.

**Figure 1:**
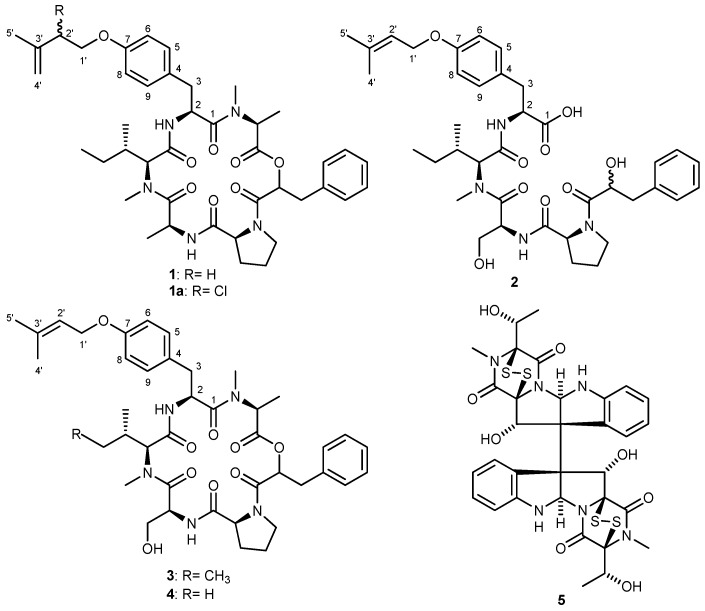
Structures of isolated compounds.

**1a** is obviously an artifact. Unfortunately, it was not possible to collect the NMR data of **1** in other deuterated solvents, since it was totally transformed to **1a**. It was interesting that the transformation to a chloro-derivative was only observed in **1** despite that compounds **3** and **4** also have double bond in the isoprenyl residue and were also measured in CDCl_3_. The structure of the new compound **1** was tentatively deduced as shown in Figure 1. 

Pullularin F (**2**) exhibited a UV/VIS spectrum with λ_max_ at 226 and 275 nm, resembling those of the previously isolated pullularins. In the HRESIMS of **2** the pseudomolecular peak at *m/z* 709.3802 [M + H]^+^ indicated the molecular formula C_38_H_52_N_4_O_9_ (calcd. for C_38_H_52_N_4_O_9_ 709.3813), hinting to the presence of only four amino acids in the molecule. The compound could not be dissolved in CDCl_3_ as for the other pullularins, but only in DMSO. Signals of the amino acid residues for proline, serine, *N*-methylated isoleucine and *O*-prenyl-tyrosine were readily observed, in the NMR spectra of **2** as well as signals for 3-phenyllactic acid, proving the absence of *N*-methylated alanine in **2**. A conspicuous feature of the NMR spectra was the upfield shift of H-2 of 3-Ph-Lac to δ_H_ 4.10 as compared to other pullularins, indicating the presence of an alcohol function instead of an ester moiety. This finding and the lack of correlations between the α-hydroxycarboxylic acid and the *O*-prenyl-tyrosine indicated that pullularin F (**2**) was a linear and not a cyclic peptide. This finding was also consistent with the molecular formula obtained from HRESIMS. Moreover, it also explained the increase in polarity of **2** in comparison to pullularins A (**3**), C (**4**) and chloro-derivative of pullularin E (**1a**).

The peptide was hydrolyzed and the stereochemistry of the amino acids analyzed also via Marfey’s method [19]. On this basis, the configurations of the amino acids in **2** were determined as L-Tyr, *N*-Me-L-Ile, L-Ser and L-Pro.

All isolated compounds were subjected to a cytotoxicity assay employing the murine lymphoma L5178Y cell line, which is summarized in Table 2. Verticillin D (**5**) showed pronounced cytotoxic activities against the tested cell line. Antiproliferative properties were also prevalent among the cyclic depsipeptides pullularin A (**3**), C (**4**) and chloro-derivative of pullularin E (**1a**) with EC_50_ values ranging between 0.1 and 6.7 µg/mL, whereas the linear pullularin F (**2**) did not exhibit any cytotoxic activity at the tested dose.

**Table 2:** EC_50_ values of the isolated compounds against L5178Y cell line.

Compound	L5178Y Survival Rate in % (10 µg/mL)	EC_50_ (µg/mL)
Chloro-derivative of pullularin E (**1a**)	15.6	5.60
Pullularin F (**2**)	114.3	>10
Pullularin A (**3**)	1.7	2.60
Pullularin C (**4**)	21.7	6.70
Verticillin D (**5**)	0.5	<0.1
Kahalalide F (positive control)		6.40

## 3. Experimental Section

### 3.1. General Experimental Procedures

Optical rotations were measured on a Perkin-Elmer-241 MC polarimeter. 1D and 2D NMR spectra were recorded on Bruker ARX 500, ARX 400 or AVANCE DMX 600 NMR spectrometers. ESIMS and HRESIMS were obtained on Finnigan LCQ Deca and Micromass Qtof 2 mass spectrometers, respectively. Solvents were distilled prior to use, and spectral grade solvents were used for spectroscopic measurements.

### 3.2. Fungal Material

Fresh, healthy leaves of *Sonneratia caseolaris* (Sonneratiaceae) were collected in September 2009 from Hainan Island of the Dongzhai Mangrove Forest. Leaves were rinsed twice with sterilized distilled water. Surface sterilization was achieved by immersing the leaves in 70% ethanol for 2 min (twice) followed by rinsing twice in sterilized distilled water. Then, the leaves were cleaved aseptically into small segments (approx. 1 cm in length). The material was placed on a Petri dish (malt agar medium) containing an antibiotic to suppress bacterial growth (medium composition: 15 g/L malt extract, 15 g/L agar, 24.4 g/L sea salt, and 0.2 g/L chloramphenicol in distilled water, pH 7.4–7.8) and incubated at room temperature (25 °C). After several days, hyphae growing from the plant material were transferred to fresh plates with the same medium, incubated again for 10 days, and periodically checked for culture purity.

### 3.3. Identification of Fungal Cultures

Fungal cultures were identiﬁed according to a molecular biological protocol by DNA ampliﬁcation and sequencing of the ITS region as described previously [20]. The sequence data have been submitted to GenBank, accession number JQ407533. The fungal strain was identiﬁed as *Bionectria ochroleuca*. A voucher strain (strain designation JCM 10.3) is kept in the Institute of Pharmaceutical Biology and Biotechnology, Duesseldorf, Germany.

### 3.4. Cultivation

Twenty Erlenmeyer ﬂasks (1 L each) containing 100 g of rice and 110 mL of distilled water were autoclaved. A small part of the medium from a Petri dish containing the puriﬁed fungus was transferred under sterile conditions to the rice medium. The fungal strain was grown on solid rice medium at room temperature (22 °C) for 40 days.

### 3.5. Extraction and Fractionation

The culture was extracted extensively with EtOAc. The EtOAc extract was taken to dryness and partitioned between *n*-hexane and 90% MeOH. The 90% MeOH fraction was chromatographed over silica gel F_254_ (Merck, Darmstadt, Germany) using gradient elution (*n*-hexane:EtOAC:DCM:MeOH). Two of the resulting fractions (VII and VIII) were chromatographed over a Sephadex LH-20 column with 100% MeOH as solvent. Based on detection by TLC (silica gel F_254_, Merck, Darmstadt, Germany) using EtOAc:MeOH:H_2_O (77:13:10) as solvent system, collected fractions were combined and subjected to semipreparative HPLC (Merck, Hitachi L-7100) using a Eurosphere 100–10 C18 column (300 × 8 mm, i.d.) with the following gradient (MeOH:H_2_O): 0 min, 10% MeOH; 5 min, 10% MeOH; 35 min 100% MeOH; 45 min, 100% MeOH. Yields of compounds were as follows: **1** (8.8 mg), **2** (2.2 mg), **3** (7.0 mg), **4** (3.4 mg), and **5** (4.2 mg).

### 3.6. Preparation and HPLC Analysis of Marfey Derivatives [21,22]

Marfey’s method was used to determine the absolute configurations of the peptides **1a**, **2**, **3** and **4**. 50 μL of 50 mM in H_2_O of each commercially available standard amino acid (D- or L-form) that is of interest was mixed with 100 µL of 1% Marfey’s reagent (FDAA = 1-fluor-dinitrophenyl-5-L-alanine amide, TCI) in acetone and heated at 40 °C for one hour. The reaction was stopped by addition of 10 µL of 2M HCl and the derivatized product dried in a freeze dryer, redissolved in MeOH and analyzed by HPLC and by LC-MS.

The isolated peptide was hydrolyzed (0.5–1 mg) with 1–2 mL 6N HCl at 110 °C for 24 h under N_2_atmosphere. The hydrolysate containing a mixture of free amino acids was cooled, dried and redissolved in water. Derivatization was achieved in the same manner as applied to standard amino acids. The retention times of the derivatized standard amino acids and of the derivatized amino acids obtained following hydrolysis of the peptide were compared to distinguish D- and L-amino acids.

Pullularin E (**1**): white powder; HRESIMS *m/z* 760.4264 (calcd for C_42_H_58_N_5_O_8_, 760.4285); Chloro-derivative of pullularin E (**1a**): white powder; [α]^20^_D_ −77 (*c* 0.5, CHCl_3_); ^1^H and ^13^C NMR in DMSO-*d*_6_, see Table 1; HRESIMS *m/z* 794.3881 [M + H]^+^ (calcd for C_42_H_56_^35^ClN_5_O_8_, 794.3896), 796.3866 (calcd for C_42_H_56_^37^ClN_5_O_8_, 796.3866).

Pullularin F (**2**): white powder; [α]^20^_D_ −140 (*c* 0.7, MeOH); ^1^H and ^13^C NMR in DMSO-*d*_6_, see Table 3; HRESIMS *m/z* 709.3802 [M + H]^+^ (calcd for C_38_H_52_N_4_O_9_, 709.3813).

**Table 3:** NMR data of pullularin F (**2**) in DMSO-*d*_6_ *^a^*, and key ^1^H–^1^H COSY, and HMBC correlations.

Position	δ_C_	δ_H_ mult. (*J* Hz)*^b^*	COSY	HMBC (H→C)
***O*-prenyl-Tyr**				
1 (C=O)	172.0 C			
2	55.4 CH	4.72 ddd (4.4, 8.8, 8.8)		1, 3, 4, C=O (*N*-Me-lle)
3	35.8 CH_2_	2.96 dd (4.0, 12.8)	2, 5	1, 4, 5
		2.59 br d (4.3)	2, 5	1, 4, 5
4	131.0 C			
5, 9	130.5 CH	7.04 d (8.6)	6, 8	3, 5, 6, 7
6, 8	116.0 CH	6.82 d (8.6)	5, 9	4
7	156.5 C			
1′	64.0 CH_2_	4.49 d (6.4)	2′	2′, 3′, 7
2′	120.5 CH	5.42 br m	1′	4′, 5′
3′	137.0 C			
4′	25.0 CH_3_	1.72 s		2′, 3′, 5′
5′	18.0 CH_3_	1.76 s		2′, 3′, 4′
NH		8.45 d (8.7)	2 (*O*-isoprenyl-Tyr)	1
***N*-Me-lle**				
1 (C=O)	168.5 C			
2	61.0 CH	4.52 d (11.0)		1, C=O (Ser)
3	31.0 CH	1.90 m *	2, 4, 3-CH_3_	3-CH_3_
4	24.0 CH_2_	1.25 m *	4b, 5	3, 3-CH_3_
		0.92 m *	4a, 5	
5	10.0 CH_3_	0.75 m *	4	3, 4
3-CH_3_	15.5 CH_3_	0.80 m *	3	2, 3, 4
*N*-CH_3_	28.4 CH_3_	2.85 s		2, C=O (Ser)
**Ser**				
1 (C=O)	171.8 C			
2	44.4 CH	4.80 m	3, NH	3, 1 (Pro)
3	62.0 CH_2_	3.65 m	2	1
		3.48 m	2	1
NH		8.18 d (4.3)	2	1
**Pro**				
1 (C=O)	171.5 C			
2	52.0 CH	4.70 m	3	3, 4
3	32.0 CH_2_	3.48 m *	2, 4	2
		3.20 m *	2, 4	
4	22.0 CH_2_	1.80 m *	3, 5	
		1.75 m *	3, 5	
5	32.0 CH_2_	1.78 m *	4, 5b	
		3.20 m *	4, 5a	
**3-Ph-Lac**				
1 (C=O)	165.4 C			
2	71.5 CH	4.10 m	3	
3	41.0 CH_2_	2.85 m *	2	1, 4, 5
		2.70 m *		1, 4, 5
4	138.0 C			
5, 9	129.0 CH	7.19 dd (2.0, 8.8) *	6, 8	4, 7
6, 8	126.0 CH	7.25 d (7.2) *	5, 9	4, 5, 6, 8
7	128.0 CH	7.26 m *		

*^a^* Measured at 600 (^1^H) and 150 (^13^C) MHz; *^b^* The assignments for methylene protons were referred as “a” in upper row, and “b” in the next row; * Overlapped signals.

### 3.7. Cell Proliferation Assay

Cytotoxicity was tested against the L5178Y mouse lymphoma cell line using the microculture tetrazolium (MTT) assay [22,23]. Experiments were repeated three times and carried out in triplicate. As negative controls, media with 0.1% (v/v) EtOH were included in all experiments.

## 4. Conclusions

Pullularins E and F (**1** and **2**) together with three known compounds (**3**–**5**) were isolated from the EtOAc extract of the endophytic fungus *Bionectria ochroleuca*. Compounds **1a**, **3**, **4**, and **5** exhibited pronounced to moderate cytotoxic activity against the mouse lymphoma cells (L5178Y) with EC_50_ values ranging between 0.1 and 6.7 µg/mL.

## Supplementary Files

Supplementary File 1: PDF-Document (PDF, 2678 KB)
