# Supplementary material for: Pullularins E and F, Two New Peptides from the Endophytic Fungus Bionectria ochroleuca Isolated from the Mangrove Plant Sonneratia caseolaris
Source: Mar Drugs. 2012 May 18;10(5):1081–91. doi: 10.3390/md10051081 (PMC3397455; doi:10.3390/md10051081)
Supplement: Supplementary File 1: — PDF-Document (PDF, 2678 KB) [file marinedrugs-10-01081-s001.pdf]

Supplementary Information

Contents

Fig 1-1 ^{1}H NMR spectrum of **1a** in DMSO-d_6....................3
Fig 1-3 ^{13}C NMR spectrum of **1a** in DMSO-d_6....................4
Fig 1-5 COSY spectrum of **1a** in DMSO-d_6....................5
Fig 1-6 HMQC spectrum of **1a** in DMSO-d_6....................6
Fig 1-8 HMBC spectrum of **1a** in DMSO-d_6....................7
Fig 1-9 ROESY spectrum of **1a** in DMSO-d_6....................8
Fig 1-10 TOCSY spectrum of **1a** in DMSO-d_6....................9
Fig 1-11 HRESIMS spectrum of **1a**....................10
Fig 2-1 HPLC chromatogram and UV-spectrum of **1**....................11
Fig 2-2 HRESIMS spectrum of **1**....................12
Fig 3-1 ^{1}H NMR spectrum of **2** in DMSO-d_6....................13
Fig 3-2 ^{13}C NMR spectrum of **2** in DMSO-d_6....................13
Fig 3-3 ROESY spectrum of **2** in DMSO-d_6....................14
Fig 3-4 HMBC spectrum of **2** in DMSO-d_6....................15
Fig 3-5 HRESIMS spectrum of **2**....................16

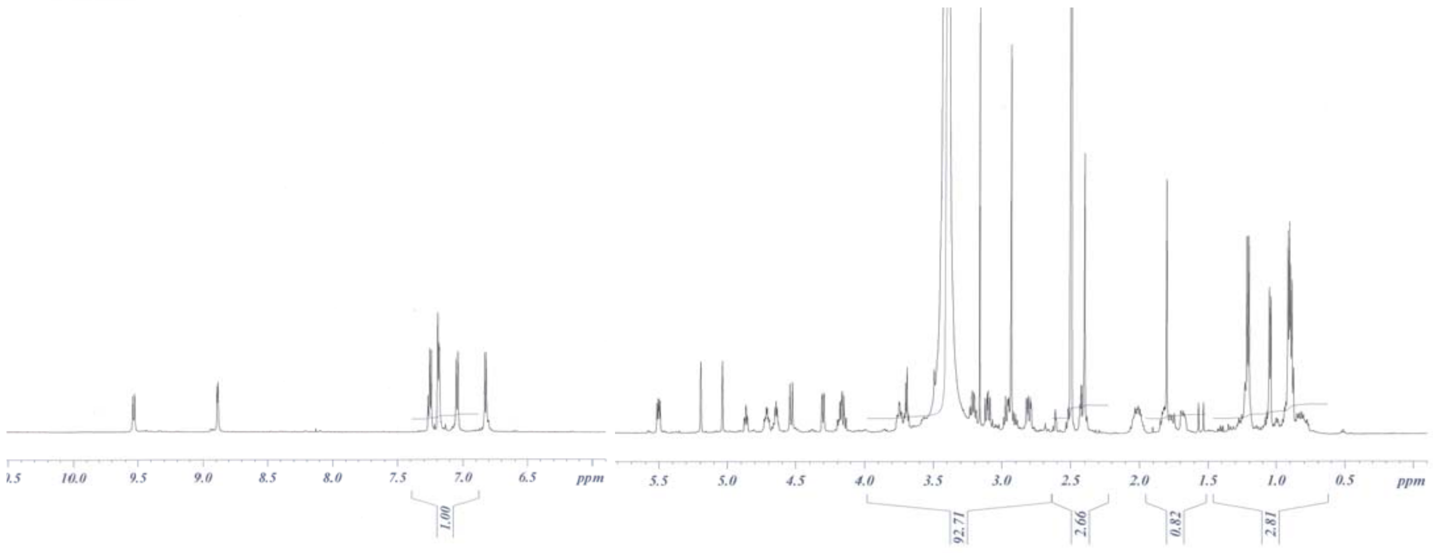

Fig 1-1 ^{1}H NMR spectrum of **1a** in DMSO-d_6

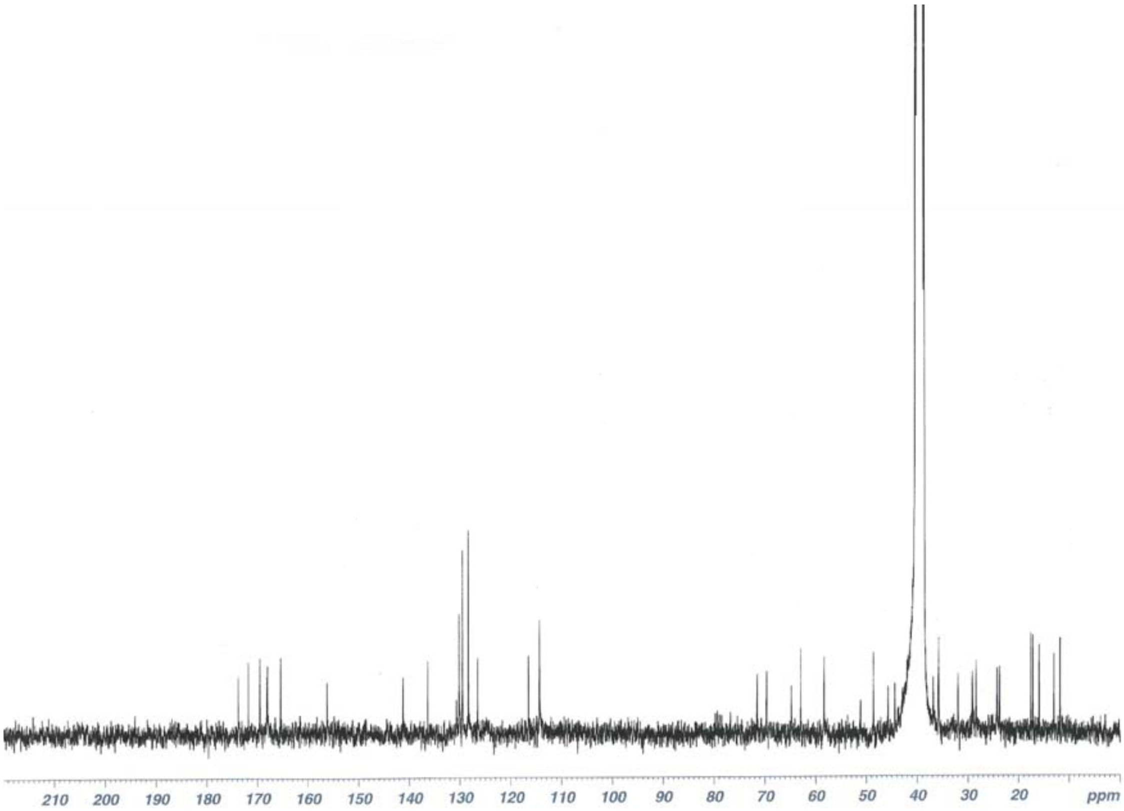

Fig 1-2 ^{13}C NMR spectrum of **1a** in DMSO-d_6

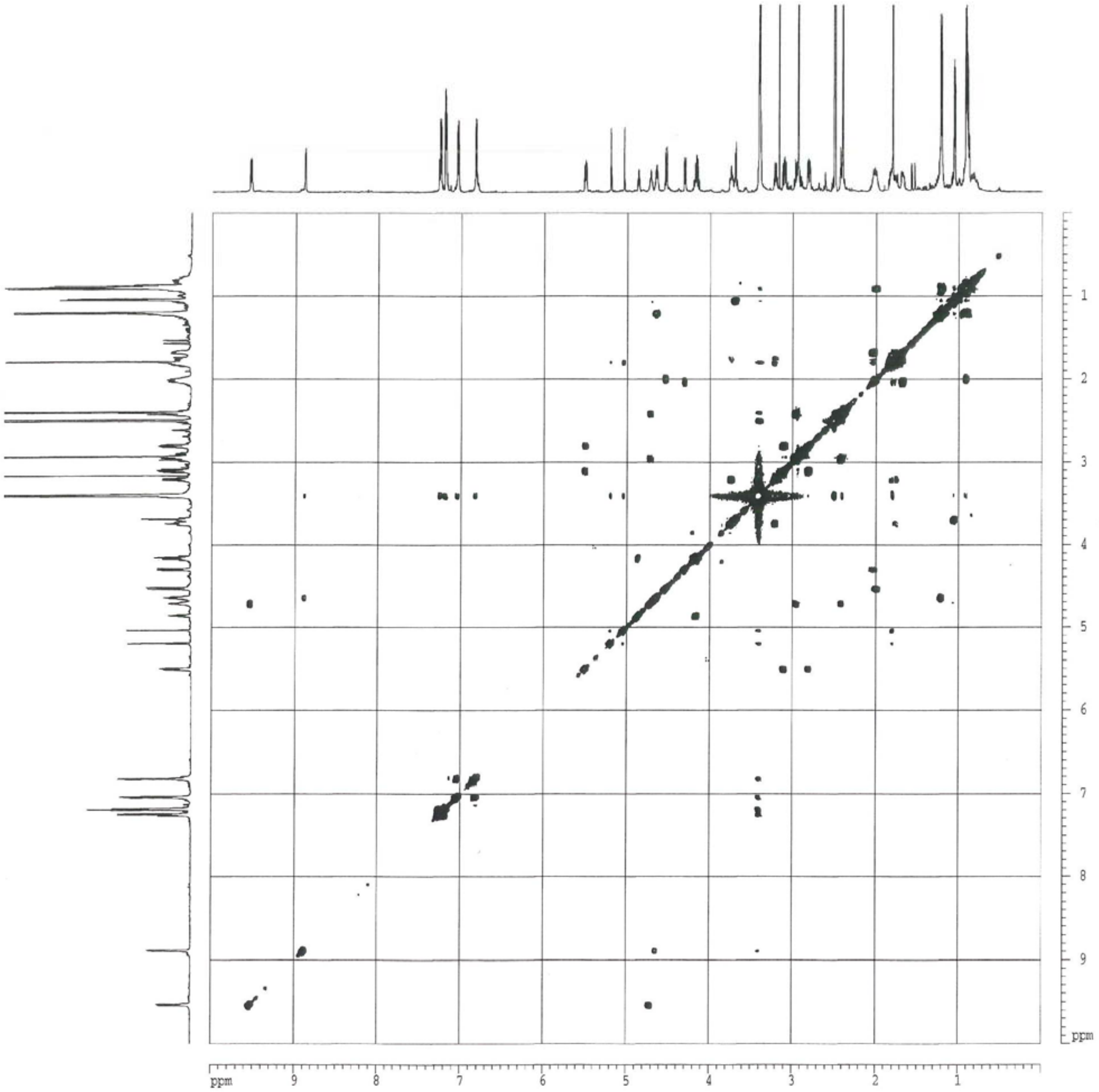

Fig 1-3 COSY spectrum of **1a** in DMSO-d_6

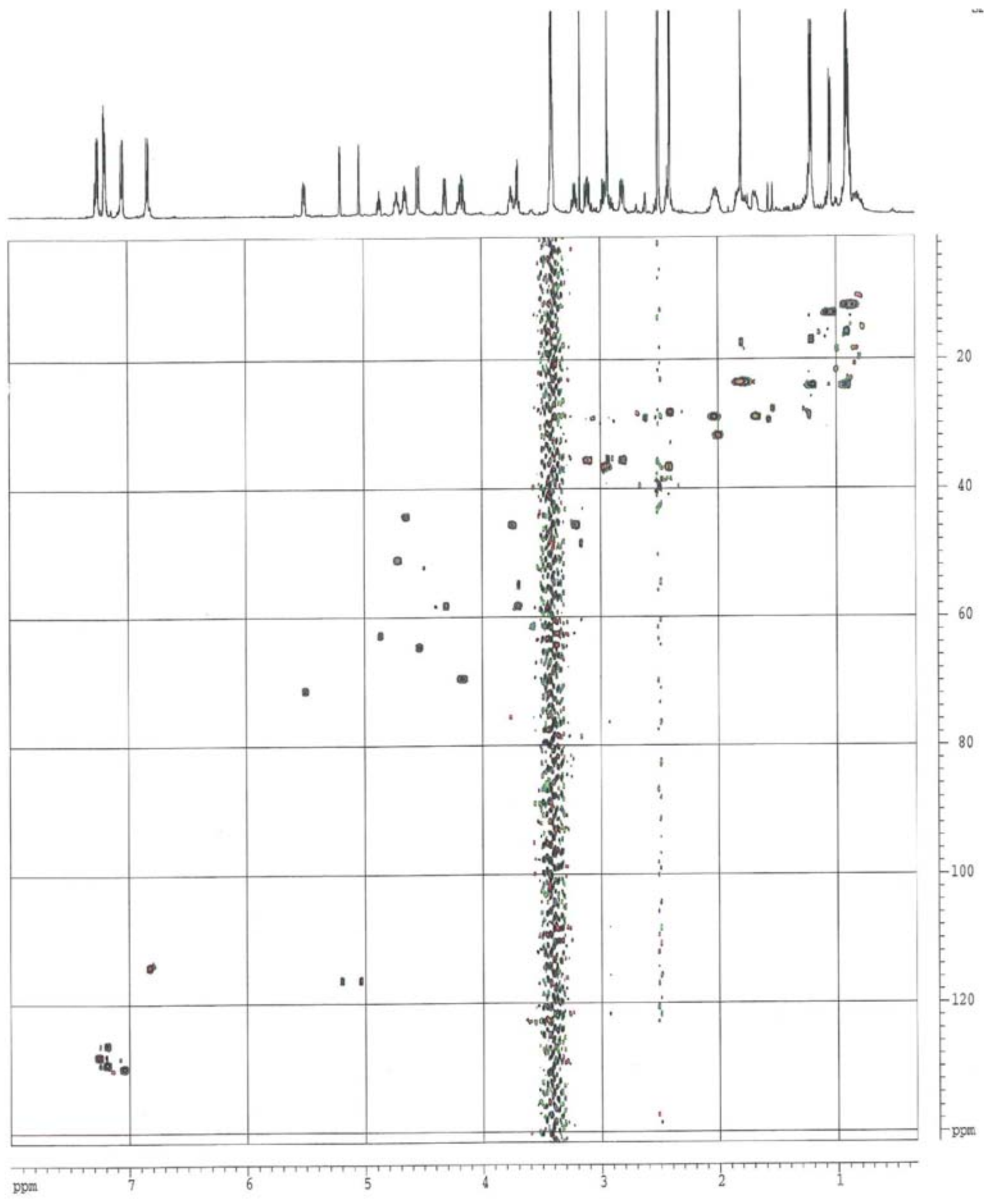

Fig 1-4 HMQC spectrum of **1a** in DMSO-d_6

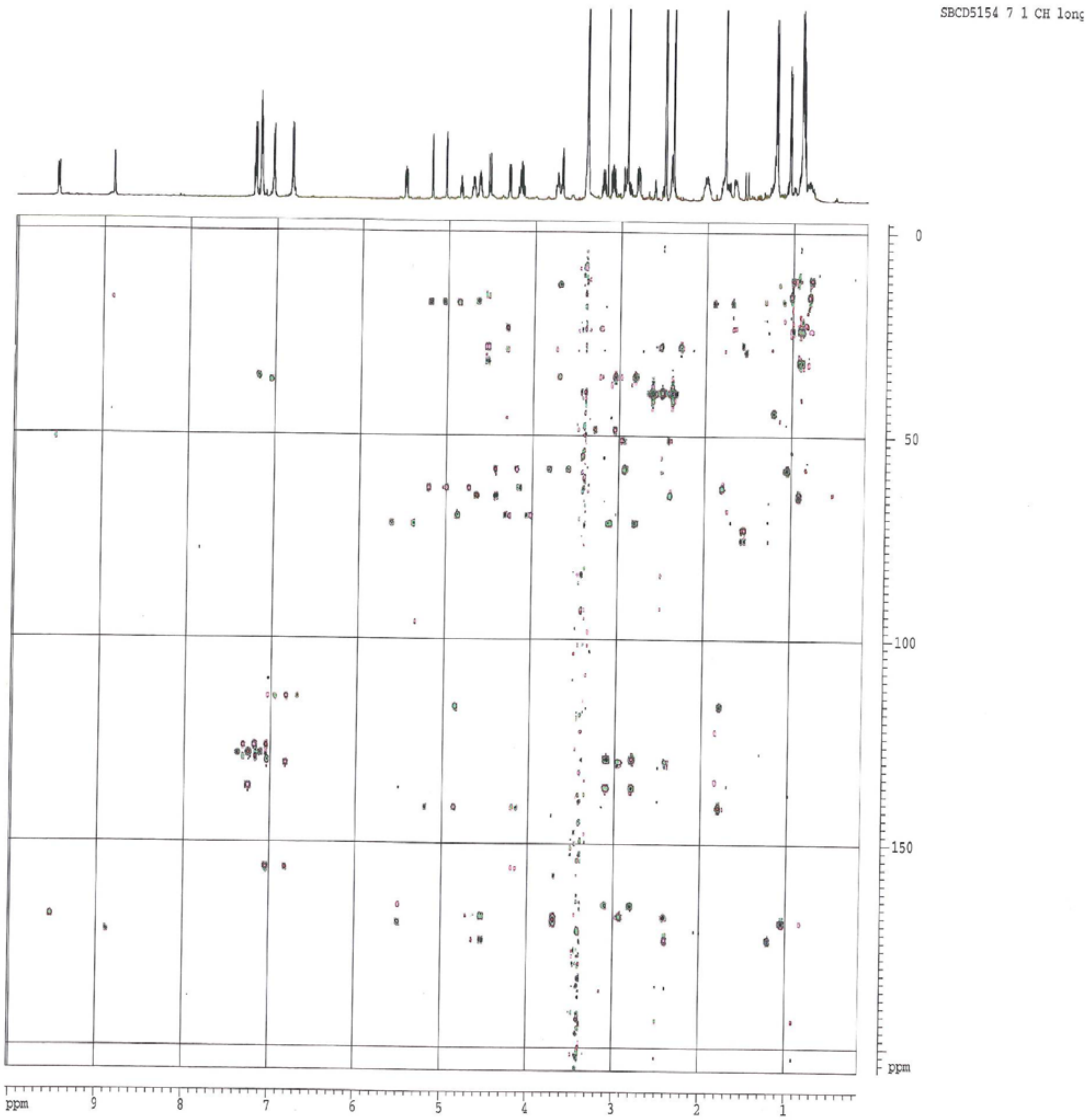

Fig 1-5 HMBC spectrum of **1a** in DMSO-d_6

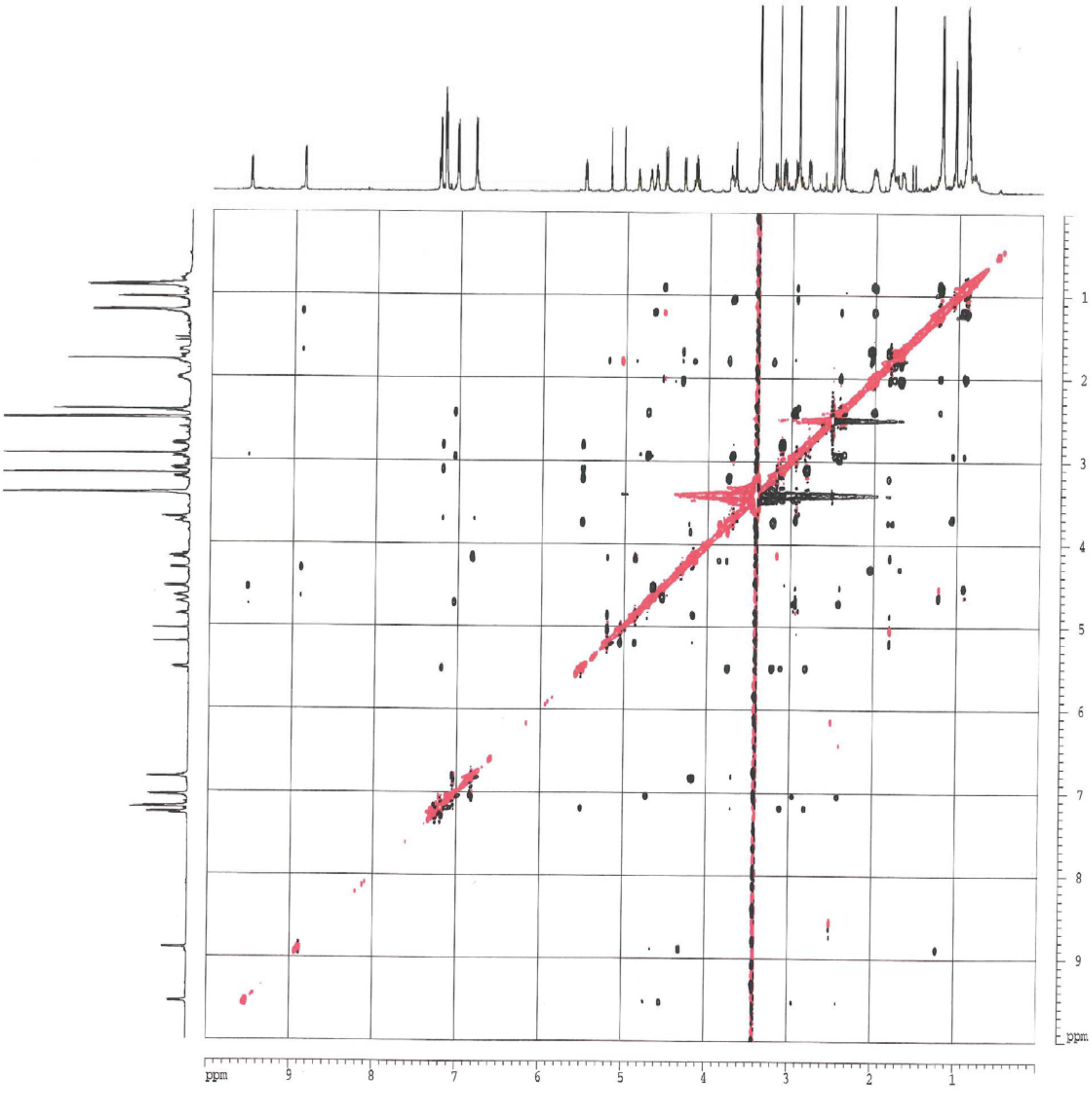

Fig 1-6 ROESY spectrum of **1a** in DMSO-d_6

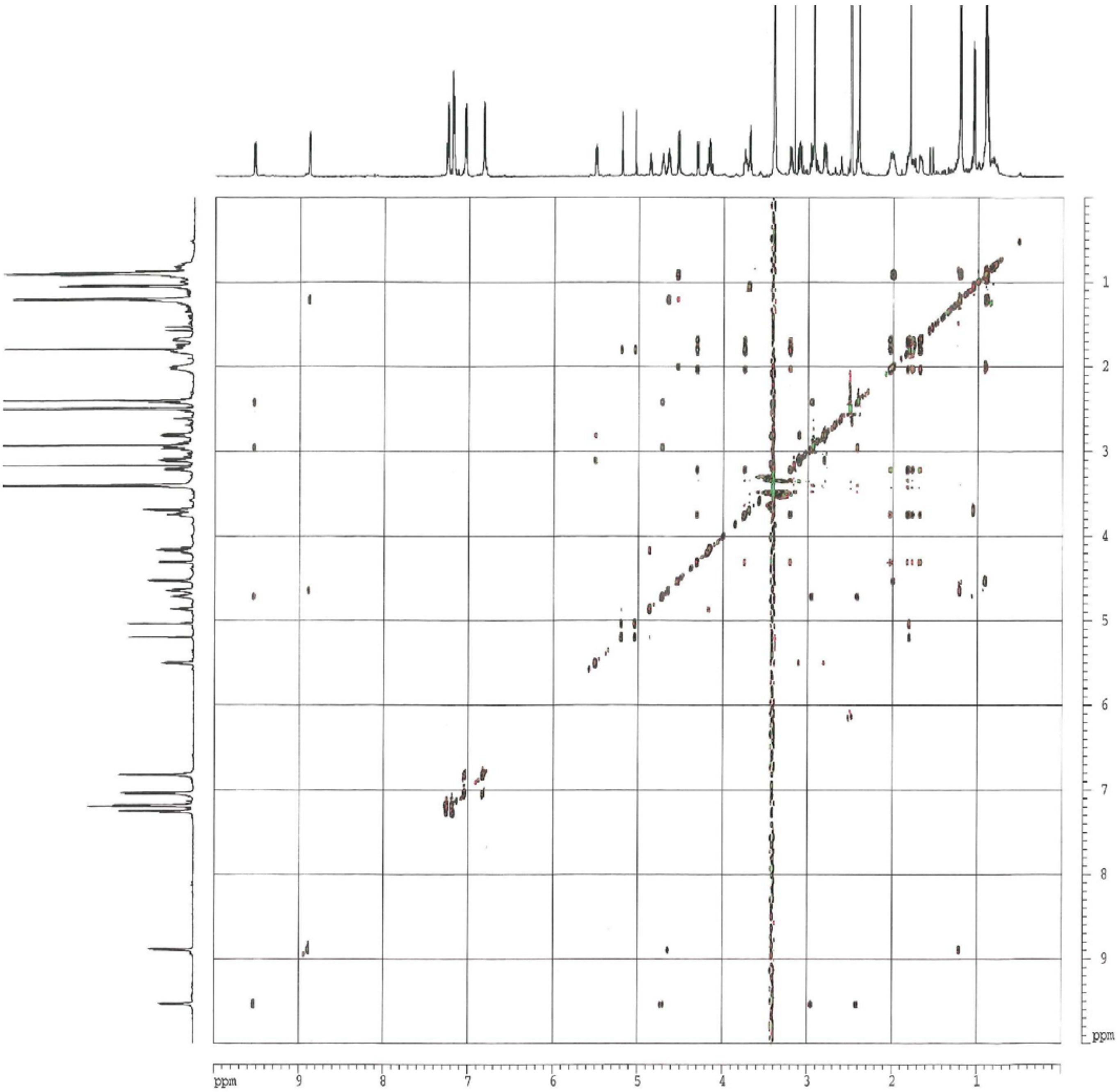

Fig 1-7 TOCSY spectrum of **1a** in DMSO-d_6

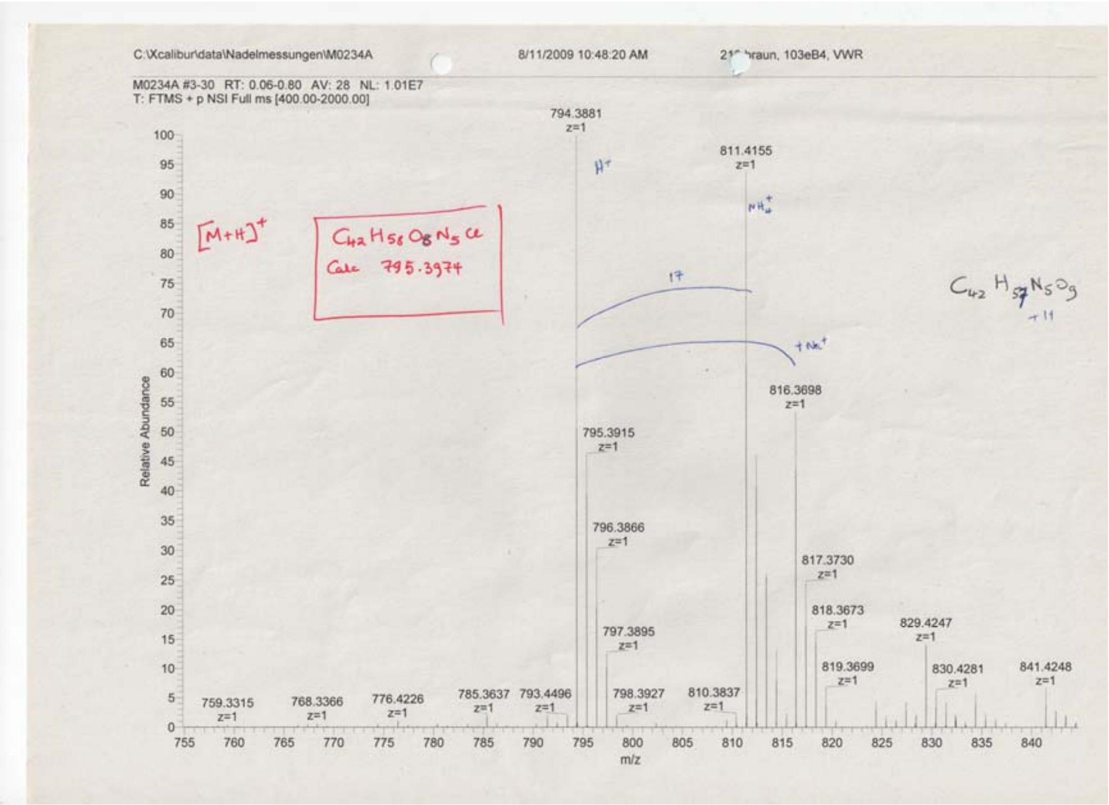

Fig 1-8 HRESIMS spectrum of **1a**

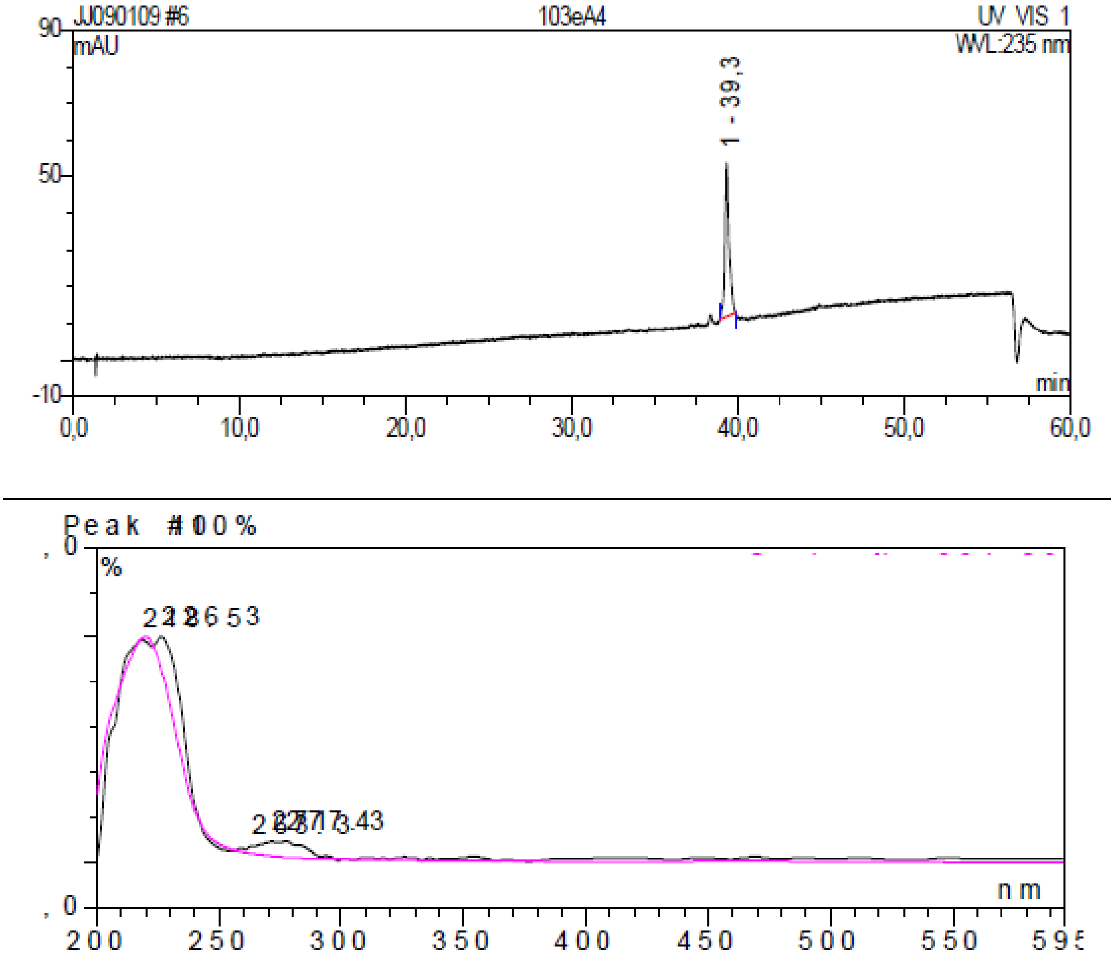

Fig 2-1 HPLC chromatogram and UV-spectrum of **1**

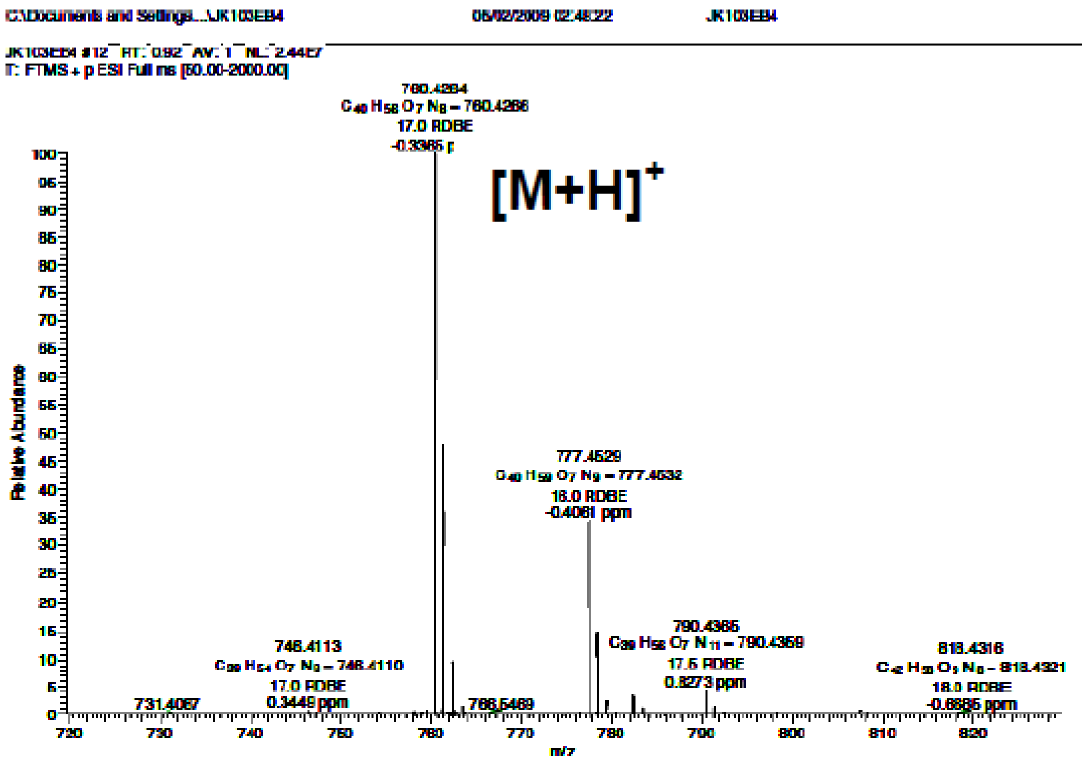

Fig 2-2 HRESIMS spectrum of **1**

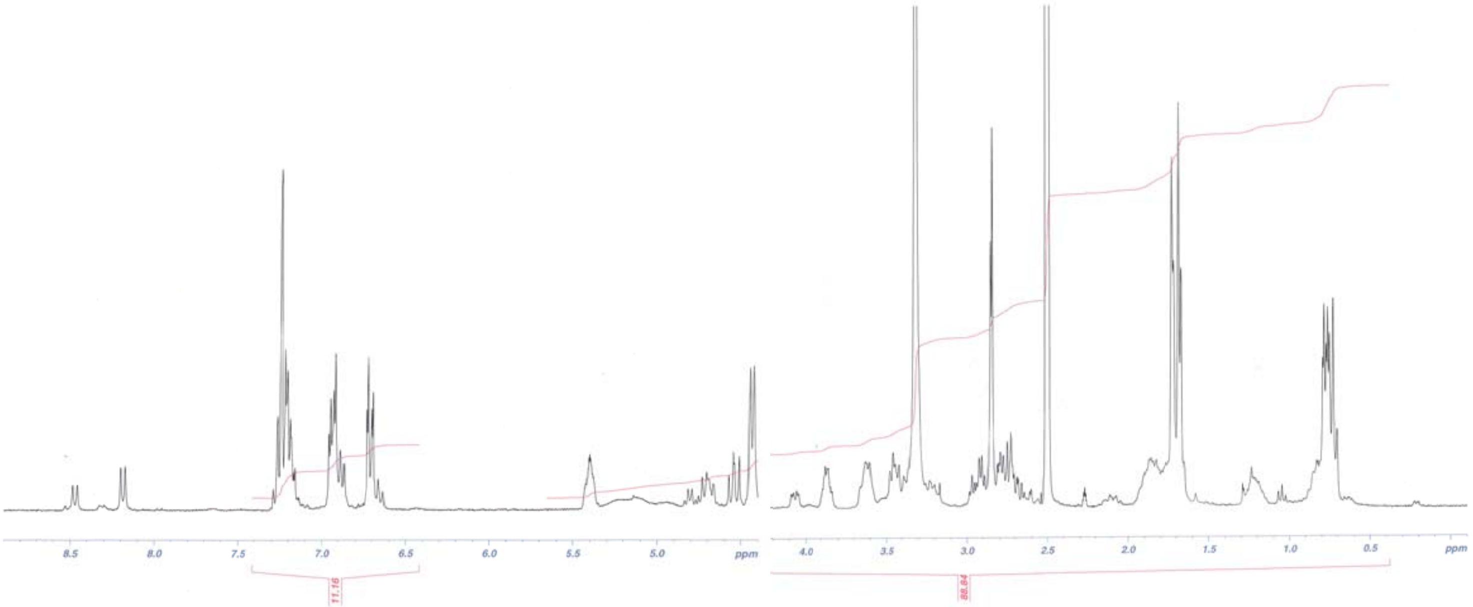

Fig 3-1 ^{1}H NMR spectrum of **2** in DMSO-d_6

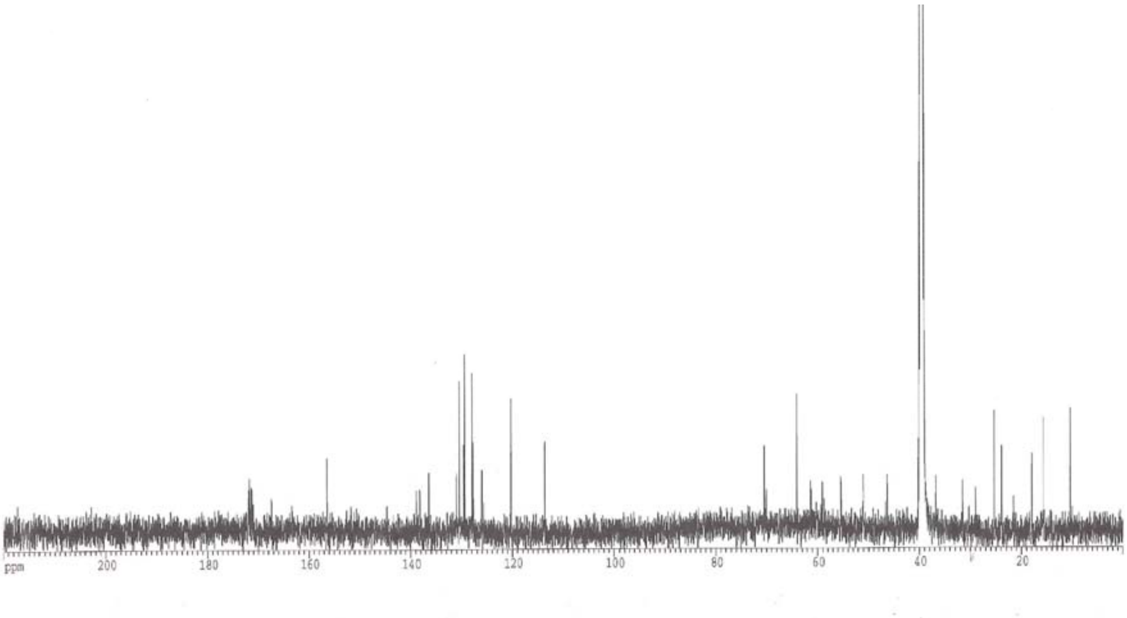

Fig 3-2 ^{13}C NMR spectrum of **2** in DMSO-d_6

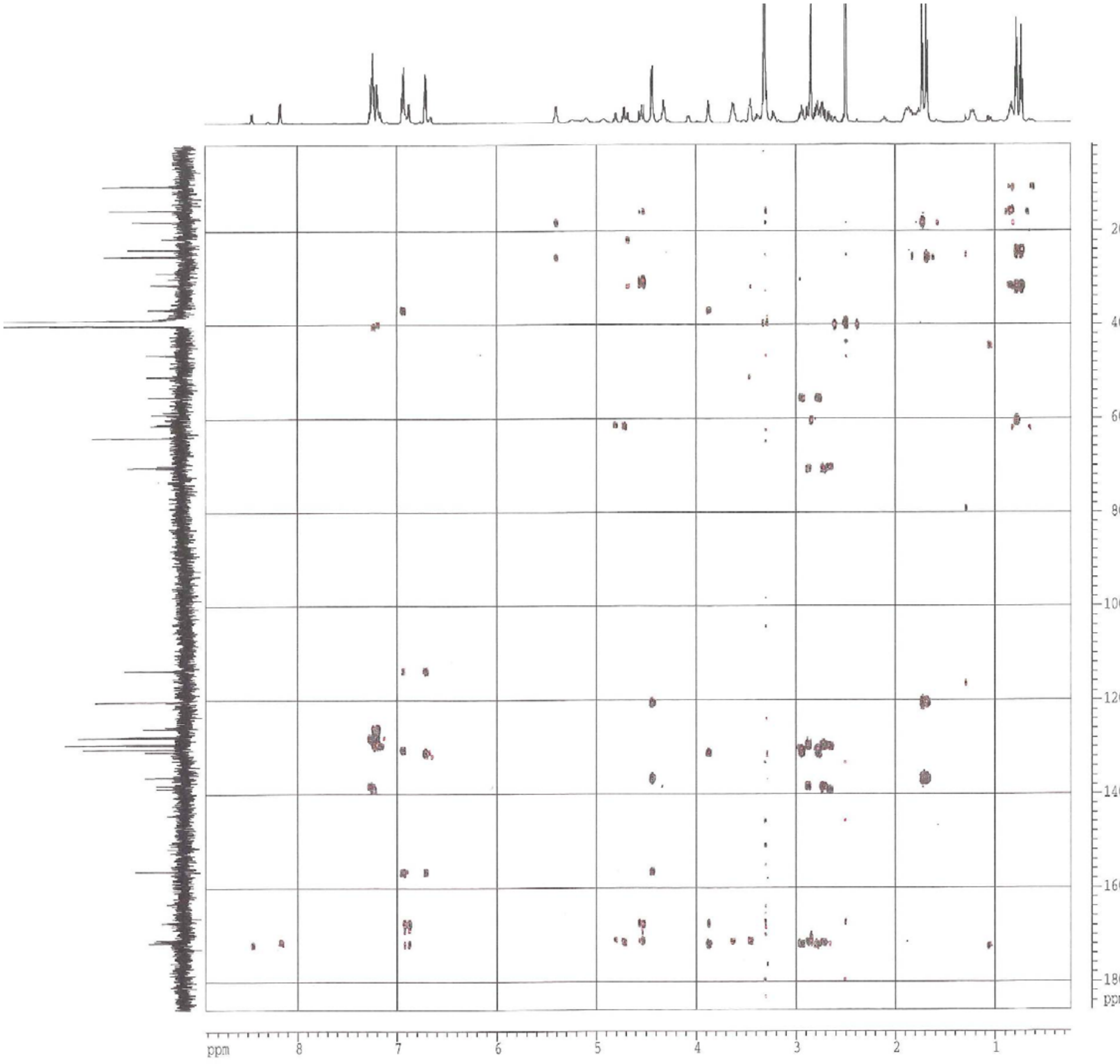

Fig 3-3 HMBC spectrum of **2** in DMSO-d_6

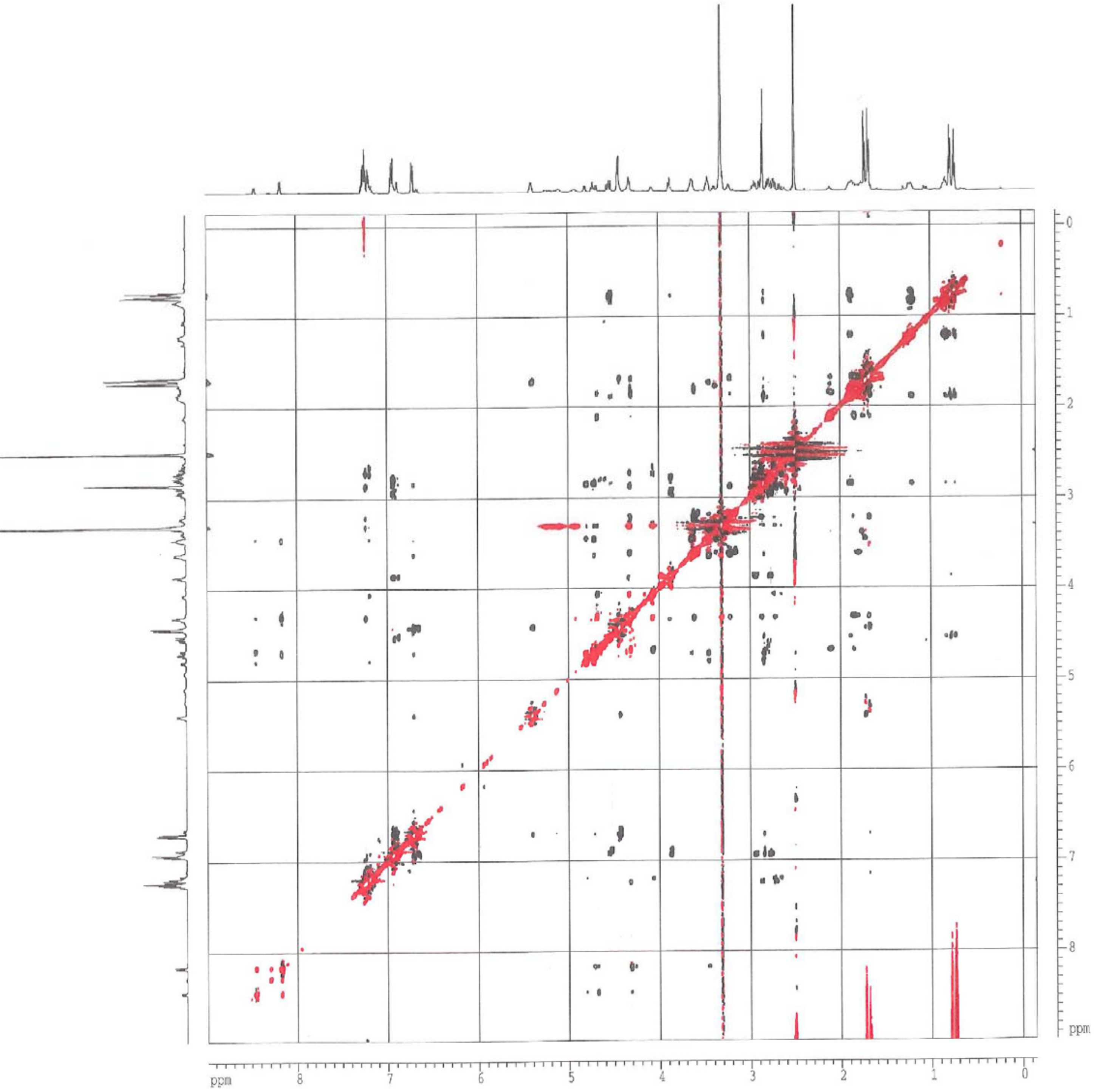

Fig 3-4 ROESY spectrum of **2**

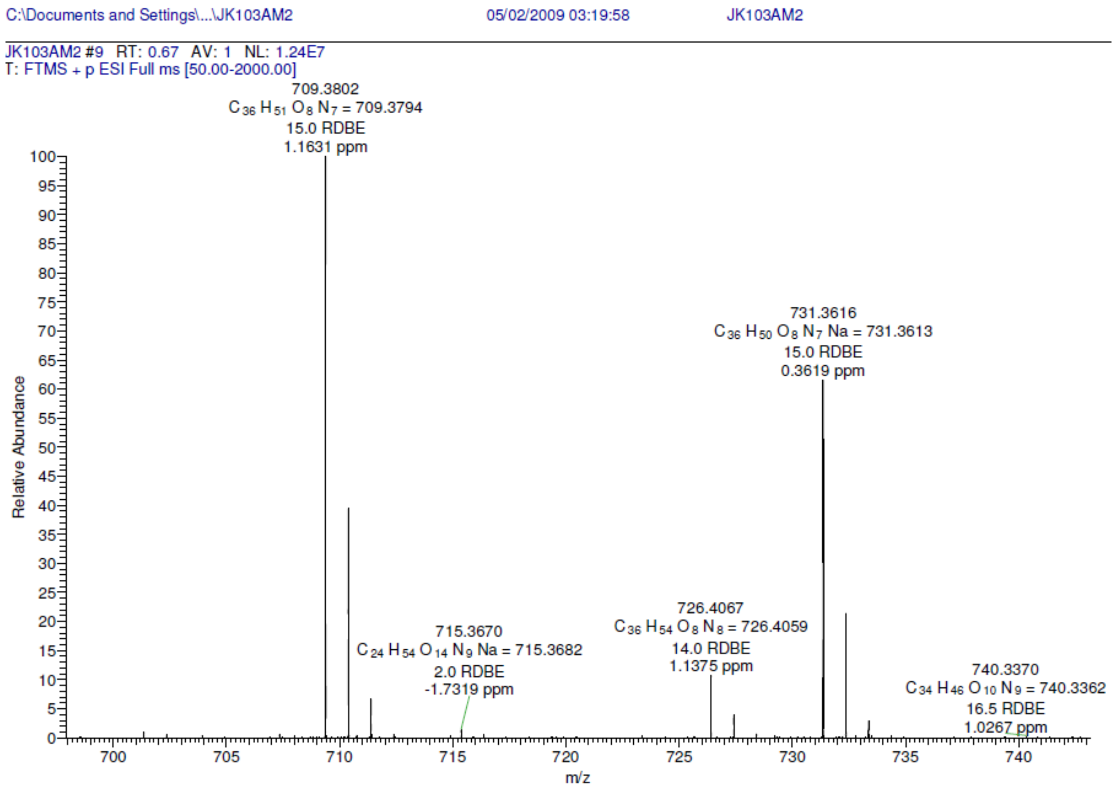

Fig 3-5 HRMS spectrum of **2**
